# Structure-Guided Design of an Engineered Streptavidin with Reusability to Purify Streptavidin-Binding Peptide Tagged Proteins or Biotinylated Proteins

**DOI:** 10.1371/journal.pone.0069530

**Published:** 2013-07-16

**Authors:** Sau-Ching Wu, Sui-Lam Wong

**Affiliations:** Department of Biological Sciences, University of Calgary, Calgary, Alberta, Canada; University of Cambridge, United Kingdom

## Abstract

Development of a high-affinity streptavidin-binding peptide (SBP) tag allows the tagged recombinant proteins to be affinity purified using the streptavidin matrix without the need of biotinylation. The major limitation of this powerful technology is the requirement to use biotin to elute the SBP-tagged proteins from the streptavidin matrix. Tight biotin binding by streptavidin essentially allows the matrix to be used only once. To address this problem, differences in interactions of biotin and SBP with streptavidin were explored. Loop_3–4_ which serves as a mobile lid for the biotin binding pocket in streptavidin is in the closed state with biotin binding. In contrast, this loop is in the open state with SBP binding. Replacement of glycine-48 with a bulkier residue (threonine) in this loop selectively reduces the biotin binding affinity (K_d_) from 4×10^−14^ M to 4.45×10^−10^ M without affecting the SBP binding affinity. Introduction of a second mutation (S27A) to the first mutein (G48T) results in the development of a novel engineered streptavidin SAVSBPM18 which could be recombinantly produced in the functional form from *Bacillus subtilis* via secretion. To form an intact binding pocket for tight binding of SBP, two diagonally oriented subunits in a tetrameric streptavidin are required. It is vital for SAVSBPM18 to be stably in the tetrameric state in solution. This was confirmed using an HPLC/Laser light scattering system. SAVSBPM18 retains high binding affinity to SBP but has reversible biotin binding capability. The SAVSBPM18 matrix can be applied to affinity purify SBP-tagged proteins or biotinylated molecules to homogeneity with high recovery in a reusable manner. A mild washing step is sufficient to regenerate the matrix which can be reused for multiple rounds. Other applications including development of automated protein purification systems, lab-on-a-chip micro-devices, reusable biosensors, bioreactors and microarrays, and strippable detection agents for various blots are possible.

## Introduction

An ideal affinity purification system for recombinant proteins should purify the target proteins from the crude extract in one step under mild conditions with high purity and excellent recovery in a cost-effective manner. Among various affinity purification systems available, biotinylated biomolecules can be efficiently purified to high purity [1], [2]. However, biotinylation of molecules either chemically [3] or enzymatically [4] requires extra steps which can be time consuming, labour intensive and costly. To overcome this problem, several streptavidin-binding peptide tags have been developed. One of them is the 38-amino-acid streptavidin-binding peptide (SBP) tag [5] that can bind streptavidin with high affinity (K_d_ ∼2.5 nM) without biotinylation. It works well whether the tag is at the N-terminal, internal or C-terminal position of the recombinant proteins [6], [7], [8]. Biotin added to the elution buffer acts as an effective competitor to elute the bound SBP-tagged proteins off. However, as streptavidin binds biotin tightly (K_d_ ∼10^−14^ M) [9], the streptavidin matrix essentially can be used only once. The use of this purification approach can be relatively costly. To vitalize this powerful purification technology, it would be ideal to have an engineered streptavidin that can bind biotin reversibly while retaining a high SBP tag binding strength. The biotin binding strength (K_d_) of this idealized streptavidin should be ∼10^−8^ M. Thus, biotin should be strong enough to displace the SBP tag from streptavidin but will not bind irreversibly to streptavidin. Consequently, the affinity matrix can be regenerated by a simple washing step under a mild elution condition and can be reused for multiple rounds. In this study, we reported the development of an engineered streptavidin designated SAVSBPM18 with these desirable properties. Its application for purification of a SBP-tagged protein and a biotinylated protein was demonstrated.

## Materials and Methods

### Construction of pSAVSBPM12 and pSAVSBPM18

The synthetic genes encoding the SAVSBPM12 and SAVSBPM18 versions of streptavidin were ordered from Epoch Biolabs Inc. (Texas, USA). These genes were cloned in an *E. coli* bluescript vector to generate pBSK-SAVSBPM12 and pBSK-SAVSBPM18, respectively. Each of these plasmids was digested by *Pst*I and *Bcl*I to release a DNA fragment encoding the streptavidin mutein. Insertion of the 442-bp SAVSBPM12 fragment to the *Pst*I and *Bcl*I digested pSSAV [10] generated pSAVSBPM12. Construction of pSAVSBPM18 was carried out by the same approach. In both SAVSBPM12 and pSAVSBPM18, P43, a strong and constitutively expressed promoter, directs the transcription. The *B. subtilis* levansucrase (SacB) signal peptide is applied for secretion.

### Production and Purification of SAVSBPM12 and SAVSBPM18

*Bacillus subtilis* WB800, an engineered 8-protease deficient strain, was used as the expression host [11]. SAVSBPM18 mutein was produced by *B. subtilis* WB800[pSAVSBPM18] cultured for 14–16 hours at 30°C in a defined medium [12]. The culture supernatant containing secreted proteins was concentrated using an Amicon ultra-15 centrifugal filter (10,000 MWCO, Millipore) and dialyzed in physiological buffered saline (PBS, 0.1 M sodium phosphate, 0.15 M sodium chloride, pH 7.5). SAVSBPM18 was purified on a biotin-agarose (Sigma, Canada) column. After loading the sample on the column, the column was washed with 5–6 column volumes of PBS. Bound proteins were eluted by PBS containing 5 mM d-biotin. Fractions containing pure SAVSBPM18 were pooled, concentrated and dialyzed against PBS. Purified SAVSBPM18 was quantified spectrophotometrically at 280 nm using a molar extinction coefficient of 41,940 M^−1^cm^−1^. Same approach was applied to cultivate *B. subtilis* WB800[pSAVSBPM12] and purify SAVSBPM12.

### Purification of β-lactamase-SBP using Immobilized SAVSBPM18 and Wild-type Streptavidin

Immobilized SAVSBPM18 was prepared by coupling purified SAVSBPM18 onto Affi-gel 15 gel (BioRad, Canada) at a concentration of 2 mg protein/ml of gel using phosphate buffer (50 mM sodium phosphate, 50 mM sodium chloride, pH 7.5) as the coupling buffer. The gel slurry was rocked for at least 4 hours at 4°C. Detailed coupling procedure and quantitation of the amount of SAVSBPM18 immobilized were according to the manufacturer’s instructions. Wild-type streptavidin (Sigma Canada) was also immobilized to Affi-15 gel in a similar manner. To demonstrate purification of ß-lactamase-SBP [13], *Bacillus subtilis* WB800[pWB980-BLA-FLSBP] (FLSBP represents the full-length 38-amino-acid streptavidin binding peptide tag) was cultured in a defined medium [12]. After washing the column with PBS, bound ß-lactamase-SBP was eluted using PBS containing 5 mM d-biotin. The column was regenerated by washing with ten column volumes of PBS.

### Purification of Biotinylated MBP using Immobilized SAVSBPM18

A mixture containing the crude soluble fraction of *E. coli* cell extract and pure biotinylated maltose binding protein (MBP-AviTag fusion, Avidity, LLC, USA) was loaded onto the SAVSBPM18 matrix. The column was washed with PBS and the bound protein was eluted by PBS containing 5 mM d-biotin. The column was regenerated by washing with ten column volumes of PBS.

### Kinetic Analysis of SAVSBPM12 and SAVSBPM18 Streptavidin Muteins

The kinetic parameters (on- and off-rates) for interactions of the muteins with biotin and SBP tag were determined in real time using the surface plasmon resonance-based BIAcoreX biosensor (GE Healthcare, Canada) as described previously [14], [15]. For the study of biotin interaction, biotinylated MBP-AviTag (Avidity, LLC) was immobilized to a CM5 sensor chip using the amine coupling method. For the study of SBP interaction, two different molecules were immobilized. The first is a synthetic peptide from Peptide 2.0 Inc. (USA). It is a 59-amino acid peptide with the sequence CGGGGSTSGGSTSGGSTSGGG**MDEKTTGWRGGHVVEGLA GELEQLRARLEHHPQGQREP** which comprises the full length SBP tag (highlighted in bold).The cysteine residue at the N-terminus is designed for thiol coupling of the peptide to the BIAcore biosensor CM5 chip. A 20-amino-acid glycine-rich linker is present between the N-terminal cysteine residue and the SBP tag to project the tag to the chip surface. The second ligand is purified β-lactamase-SBP. This protein was coupled to the CM5 sensor chip via the amine coupling approach. All the coupling procedures (thiol and amine coupling) were performed according to the manufacturer’s instructions. A blank flow cell was used as the reference cell in all studies. Kinetic parameters (k_a_ and k_off_) were determined using BIAevaluation software provided by the manufacturer.

Since SAVSBPM12 mutein retains a relatively high binding affinity towards biotin, this mutein purified from the *B. subtilis* culture supernatant via biotin-agarose was subject to the denaturation and renaturation cycle [16] to ensure the removal of any tightly bound biotin in this protein. The successfully refolded muteins were affinity purified by iminobiotin-agarose [17] and used for the biosensor study.

### Absolute Molecular Mass Determination of SAVSBPM18 using HPLC

The molecular mass and molecular size distributions of SAVSBPM18 were determined by a multi-angle light scattering (MALS) detection system used in conjunction with the size exclusion chromatography (SEC) [18]. A pure sample of SAVSBPM18 was fractionated on a size exclusion column (TSK-GEL SuperSW2000, Tosoh Bioscience, USA) using a Shimadzu Prominence HPLC system which is equipped with a column temperature control oven (Shimadzu Prominence CTO-20AC Column Temperature Oven) to control the temperature of both the injector and column. The light scattering instrumentation consists of a multi-angle, static light scattering detector (DAWN-HELEOS II from Wyatt Technology Corp., USA) interfaced with a dynamic light scattering detector (WyattQELS) and an online concentration detector (Wyatt Optilab rEX differential refractometer). Absolute molecular mass, molecular size and fraction distributions of SAVSBPM18 were analyzed using ASTRA V software (Wyatt Technology Corp., USA).

### Analysis of Interactions between SBP Tag and Streptavidin using Molegro Viewer

**Figure 1:**
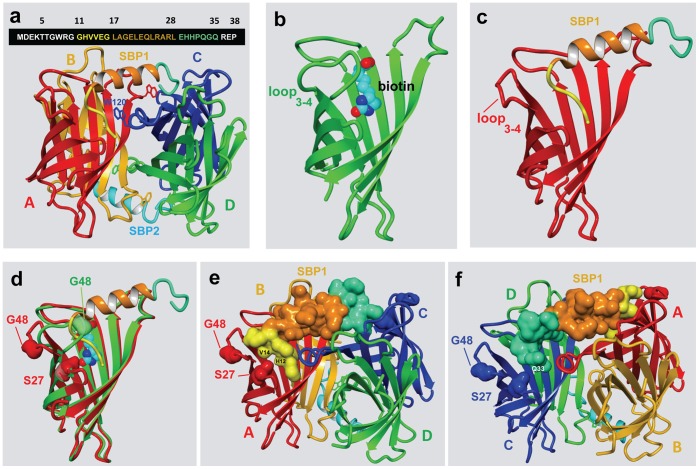
Interactions between SBP tags and streptavidin. a: Sequence of the 38 amino-acid SBP tag and the streptavidin-SBP complex. The SBP sequences in white are not visible in the streptavidin-SBP tag complex (4jo6). The N- and C-terminal residues involved in binding to the SBP tag binding pocket in streptavidin are colored in yellow and greenish blue. The spacer sequence linking both the N- and C-terminal streptavidin binding peptides is shown in brown color. One tetrameric streptavidin can bind two SBP tags. The four streptavidin subunits are colored red (A subunit), orange (B subunit), blue (C subunit) and green (D subunit). A complete SBP-binding pocket in the A subunit requires Trp-120 from the C subunit. Trp-120 residue in each subunit is illustrated. b and c: a single subunit in tetrameric streptavidin binds biotin and SBP tag, respectively. d: Superimposition of the structures shown in b and c. Both the S27 and G48 residues in these structures are shown. e: Binding of the N-terminal SBP tag (yellow) in the A subunit of a tetrameric streptavidin. The entire SBP tag is shown in the space filled model. S27 and G48 in streptavidin and H12 and V14 in the N-terminal SBP tag are labeled. Only S27 (but not G48) makes close contacts with H12 and V14. The subunits of the tetrameric streptavidin protein are colored as specified in panel a. f: Binding of the C-terminal SBP tag (greenish blue) in the C subunit. G48 in streptavidin does not make any close contact with the C-terminal SBP tag. S27 makes weak contact with Q33 in SBP.

The pdb file (4jo6) of the streptavidin-SBP tag complex was used as the starting file. This file was imported to Molegro molecular viewer (Version 2.5.0) [19] by clicking the file menu in the program and selecting “import molecules” from the submenu. In this pdb file, the four streptavidin subunits and the two bound SBP tags were labelled as chains A, B, C, D, Y and Z, respectively. Chain Y of SBP is the peptide tag that binds to subunits A and C of streptavidin. In this study, chain Y was selected as a ligand by right clicking the name of this chain displayed in the workplace explorer window and selecting “convert protein to ligand” under the submenu. To estimate the interaction free energy between the SBP ligand (chain Y) with streptavidin, the ligand energy inspector module was selected under the tools menu in this program. Before analysis, both the ligand and protein hydrogen bonding positions were optimized and the ligand was energy minimized using the action panel in the ligand energy inspector module. The protein-ligand interaction free energy [E_inter_, sum of the steric interaction energy, hydrogen bonding energy, short- (r <4.5 Å) and long-range (r >4.5 Å) electrostatic interaction energies] was expressed in the form of the MolDock score [19] in arbitrary units. Stepwise operation of the program and the details of the MolDock score can be found in the Molegro molecular viewer user manual and the tutorial videos that are available from the Molegro website (http://www.clcbio.com/products/molegro/). The graphic drawings of the structure of SBP-SAVSBPM18 and streptavidin-biotin (1SWE) shown in Fig. 1 were generated by using the Yasara [20] (YASARA Biosciences GmbH, Austria) program.

## Results

### Rational Design of Streptavidin SAVSBPM18

Streptavidin is a homo-tetrameric protein with a biotin binding site in each subunit [9]. It binds biotin with high affinity (K_d_ ∼10^−14^ M) through extensive hydrogen bonding, hydrophobic interactions and closure of a mobile loop [21], [22]. The mechanism for the tight binding between SBP tag and streptavidin has also been examined recently by determining the structure of the SBP-streptavidin complex via X-ray crystallography [13]. Out of 38 residues in the full-length SBP tag, only 25 amino acids (residues 11–35 colored in Fig. 1a) form a well-ordered structure that can be visualized in the streptavidin-SBP tag complex. Further truncation of the last residue (residue 35 numbered according to the full-length SBP tag) of the 25-amino-acid SBP sequence leads to the generation of a shorter tag designated SBP-Tag2 which retains comparable binding strength as the full-length SBP tag [13]. This 24-amino-acid sequence can be divided into three functional segments. The first six amino acids (GHVVEG) and the last six amino acids (EHHPQG) from the tag form two binding segments. Each segment binds to a binding pocket formed by two different subunits in the tetrameric streptavidin. The N-terminal binding segment binds to a binding pocket formed mainly by the A subunit. Trp-120 residue from the C subunit is required to form a complete binding pocket (Fig. 1a). Similarly, the C-terminal binding peptide binds to the binding pocket formed mainly by the C subunit with Trp-120 residue from the A subunit being required to form a complete binding pocket. The 11-amino-acid central segment has dual functions. First, it forms a helical structure which functions as a spacer with appropriate length to allow the two binding segments to insert to the peptide binding pockets in streptavidin. At the same time, some of the residues (e.g. Leu residues in positions 17, 21, 24 and 28) also interact with streptavidin to strengthen the binding. In essence, each of the SBP tags acts like a staple pinning into the two diagonally arranged peptide (or biotin) binding pockets (A/C or B/D) in a tetrameric streptavidin (Fig. 1a, e and f).

To develop an engineered streptavidin (SAVSBPM18) with high affinity to SBP but lower affinity to biotin, it is crucial to identify streptavidin residues that are only important for biotin binding but are not critical for SBP binding for mutagenesis. Since the two streptavidin binding peptide segments from the SBP tag essentially bind to the biotin binding pockets (Fig. 1, panels a and d), most of the streptavidin residues that are critical for biotin binding are also important for SBP binding. However, one major difference exists between biotin binding and SBP tag binding in streptavidin. It is the conformation of loop_3–4_ (residues 45–53) which connects the β-strands 3 and 4 in streptavidin (Fig. 1, panels b and c). Loop_3–4_ in streptavidin normally functions as a gateway controlling the entry and exit of biotin [22]. In the absence of biotin, loop_3–4_ is dynamic and is mainly in the open configuration. Biotin can freely access to the biotin binding pocket. With biotin binding, three residues (Val-47, Gly-48 and Asn-49) in loop_3–4_ make major contacts with biotin. Loop_3–4_ then adopts a closed configuration (Fig. 1b) [22] and essentially traps biotin in the biotin binding pocket. In contrary, loop_3–4_ remains in an open configuration after binding of the SBP tag (Fig. 1c). Gly-48 in the loop interacts with biotin but is clearly not involved in SBP binding (Fig. 1, panels d-f). Replacement of Gly-48 with Thr should affect how this loop makes contact with biotin. Since threonine has a bulkier side chain than glycine, residues Val-47 and Asn-49 may not be able to make close contacts with biotin anymore. As glycine is crucial to induce the turn formation in a loop [23], the replacement residue should also have a high propensity for turn formation. Threonine meets this requirement [24]. The G48T streptavidin mutein designated SAVSBPM12 was constructed and produced from *B. subtilis* via secretion. This mutein retained almost full binding strength to the SBP tag (Table 1) but had a lower biotin binding affinity (K_d = _4.45×10^−10^ M). Kinetic studies indicated that the drop in biotin binding affinity was mainly contributed by a significant increase (>10^3^ times) in off-rate which is consistent with the prediction that the mutated loop_3–4_ is no longer able to make good contact with the bound biotin to retain it in the biotin binding site. Although the drop in biotin binding affinity in SAVSBPM12 is significant (10^4^ times in reference to wild-type streptavidin), it is not sufficient for SAVSBPM12 to bind biotin in a reversible manner. One extra mutation which has minimal effect on SBP binding would be needed to further reduce the biotin binding affinity.

**Table 1:** Kinetic parameters for the interactions between streptavidin variants and the binding ligands.

	SAVSBPM18			wt SAV			SAVSBPM12		
Binding ligand	On-rate (M ^−1^s^−1^)	Off-rate (s^−1^)	K_d_ (M)	On-rate (M ^−1^s^−1^)	Off-rate (s^−1^)	K_d_ (M)	On-rate (M ^−1^s^−1^)	Off-rate (s^−1^)	K_d_ (M)
Biotinylated MBP	2.30×10^5^	2.65×10^−3^	1.15×10^−8^	5.13×10^6^ *	2×10^−7^ ***	4×10^−14^ **	1.10×10^6^	4.89×10^−4^	4.45×10^−10^
SBP tag (peptide)	1.07×10^5^	8.21×10^−4^	7.67×10^−9^	3.00×10^5^	4.96×10^−4^	1.65×10^−9^	2.81×10^5^	5.60×10^−4^	1.99×10^−9^
β-lactamase-SBP	4.53×10^4^	7.11×10^−4^	1.57×10^−8^	9.50×10^4^	8.16×10^−4^	8.59×10^−9^	ND	ND	ND

* Value reported by Qureshi and Wong [52].

** Value reported by Green [9].

*** Value calculated based on the on-rate and K_d_ shown in the table.

ND: Not determined.

The interactions between the SBP tag and streptavidin were quantitatively estimated using the ligand energy inspector module in Molegro molecular viewer [19]. This module tabled all the residues in streptavidin that interact with SBP with their interaction strengths expressed in terms of the MolDock scores [19]. Top five residues in streptavidin making the strongest interactions with a bound SBP tag (Chain Y in 4jo6) are listed in Table 2. A more negative value reflects a stronger interaction. Ser-27 was identified as an attractive candidate for mutation because it interacts weakly with SBP (Table 2). Since the sequences in the N- and C-terminal binding segments of the SBP tag are different, they interact with the binding pocket differently. Ser-27 in the A subunit of streptavidin interacts weakly with His-12 and Val-14 in the N-terminal binding segment of SBP (Fig. 1e). The same is true for Ser-27 in the C subunit which also interacts weakly with Q33 in the C-terminal segment (Fig. 1f). The sum of these Ser-27 specific interaction free energy scores (−3.95) corresponds to only 1.02% of the total interaction free energy score (−388) for one SBP tag to bind to streptavidin (Table 2). If this estimation is correct, the S27A mutation should not dramatically reduce the binding affinity of the SBP tag to streptavidin. In contrast, the S27A mutation [16] has previously been shown to change the biotin dissociation constant from 10^−14^ M to 10^−12^ M. Therefore, SAVSBPM12 was further modified to generate SAVSBPM18 which has double mutations (S27A, G48T).

**Table 2:** Comparison of interaction free energy of Ser-27 in streptavidin (SAV)-SBP complex (chain Y in 4jo6) with that of top five residues in SAV which are making the strongest interaction with the same SBP tag.

SAV subunit	SAV residue	Interaction free energy*with SBP	SAV-SBP interactionfree energy*	Percentage of total interactionfree energy
C	Trp-120	−40.10	−388.0	10.3
A	Trp-120	−33.95	−388.0	8.75
C	Trp-79	−29.88	−388.0	7.70
A	Lys-121	−28.86	−388.0	7.44
C	Lys-121	−24.76	−388.0	6.38
A	Ser-27	−2.78	−388.0	0.72
C	Ser-27	−1.17	−388.0	0.30

Chain Y of SBP only makes contact with subunits A and C in streptavidin.

* The interaction free energy is expressed in terms of the MolDock score. SAV-SBP interaction free energy represents the total interaction free energy for the interactions between streptavidin (subunits A and C) with one of the bound SBP tags (chain Y).

### Binding Properties of SAVSBPM18

**Figure 2:**
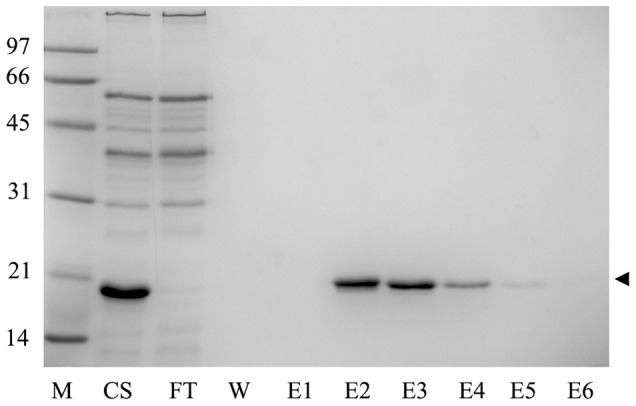
Purification of SAVSBPM18. Secreted SAVSBPM18 produced from WB800[pSAVSBPM18] was affinity purified using biotin-agarose. Fractions were analyzed by SDS-PAGE. M: Molecular weight markers. The numbers shown on the left represent the molecular mass expressed in kDa; CS: culture supernatant; FT: Flow-through fraction; W: wash fraction; E1–E6: elution fractions. Arrowhead marks the position of SAVSBPM18.

**Figure 3:**
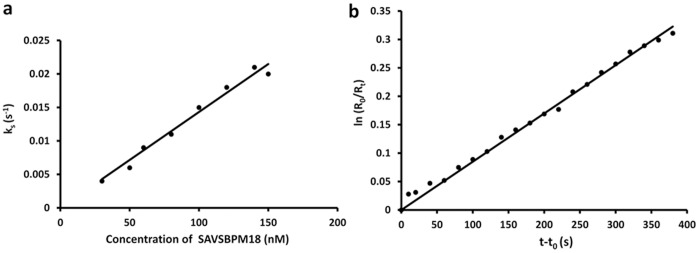
Determination of kinetic parameters of the interaction between SAVSBPM18 and the synthetic SBP tag. SBP tag was immobilized to the sensor chip and SAVSBPM18 served as the analyte. k_on_ (panel a) and k_off_ (panel b) (slopes of the plots) were estimated using BIAevaluation software.

Streptavidin SAVSBPM18 was produced from *B. subtilis* via secretion in its functional form with a production yield of around 10 mg/liter. The mutein was affinity purified to homogeneity using biotin-agarose (Fig. 2) with a recovery of 90%. The binding strength of the purified SAVSBPM18 to SBP tags immobilized on the sensor chip was determined by the surface plasmon resonance based BIAcore biosensor. A typical determination of k_on_ and k_off_ by this method was shown in Fig. 3. Although the mutein showed a lower binding affinity, its binding strength (K_d = _7.67×10^−9^ M, Table 1) has the same order of magnitude as that of wild-type streptavidin (1.65×10^−9^ M). To examine whether fusing the SBP tag to a protein affects its interaction with streptavidin, a β-lactamase-SBP fusion [13] was used in this study. This fusion has a 19 amino-acid linker as the spacer and SBP is at the C-terminal end. Comparing the dissociation constants of wild-type streptavidin against SBP tag and SBP tagged-β-lactamase indicated that fusing the SBP tag to β-lactamase did reduce the binding affinity by approximately 5-fold. However, the fusion protein still retained the nanomolar binding affinity towards wild-type streptavidin. With β-lactamase-SBP immobilized to the sensor chip for analysis, SAVSBPM18 showed a 1.8-fold reduction in binding affinity (K_d_ = 1.57×10^−8^ M) in reference to wild type streptavidin. In terms of the biotin binding affinity, SAVSBPM18 had a K_d_ of 1.15×10^−8^ M (Table 1). In fact, SAVSBPM18 binds to β-lactamase-SBP and biotinylated maltose binding protein (MBP) with similar affinities. Biotin in excess should be an effective competitor to displace SBP off from SAVSBPM18.

### Tetrameric State of SAVSBPM18

**Figure 4:**
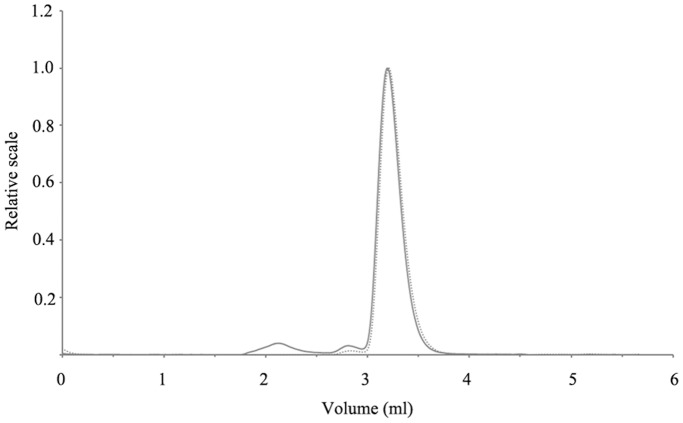
Tetrameric state of SAVSBPM18 as analysed by HPLC size exclusion chromatography with in-line static and dynamic light scattering detectors, UV monitor and refractometer. Purified SAVSBPM18 at room temperature in the absence of biotin was applied to a size-exclusion column. Solid line: elution profile monitored by the static light scattering detector. Dotted line: elution profile monitored by the UV detector. Of the two UV detectable peaks (elution volume ∼2.8 ml and 3.2 ml, respectively), the major peak (eluted around 3.2 ml) represents the tetrameric SAVSBPM18 with a molecular mass of 65 kDa. The mass fraction of this peak is 99.3%.

For tight binding to the SBP tag, SAVSBPM18 has to be in the tetrameric state (Fig. 1). The oligomeric state of SAVSBPM18 in solution in the absence or presence of biotin at room temperature was analyzed using the high-performance size-exclusion chromatography combined with in-line dynamic and static light scattering detectors, refractometer and UV monitor [18]. In the absence of biotin, a single major peak with a molecular mass of 64.5 kDa and a mass fraction of 99.3% was detected in the chromatogram (Fig. 4). The polydispersity index of this peak (1.007) reflects that SAVSBPM18 in this peak has a uniform size, shape and mass distribution. Since the expected molecular mass of the tetrameric SAVSBPM18 is 66 kDa, this demonstrates that SAVSBPM18 in solution is in the tetrameric state. No protein peaks corresponding to dimeric and monomeric SAVSBPM18 subunits were detected. In the presence of biotin, a single protein peak with a mass fraction of 99% was observed in the chromatogram. The molecular mass of the SAVSBPM18-biotin complex was determined to be 69.6 kDa with a polydispersity index of 1.013. To confirm the tetrameric nature of SAVSBPM18 at a higher temperature, SAVSBPM18 was placed in a 40°C water bath for 30 minutes and subsequently injected to HPLC with the temperature of both the injector and the column controlled at 40°C. The chromatogram once again showed a single peak of tetrameric SAVSBPM18 with a mass fraction of 99% either in the absence or presence of biotin (data not shown). The molecular mass was 64.2 kDa in the absence of biotin and 65.7 kDa in the presence of biotin, respectively. Since protein purification in general is performed at either room temperature or 4°C, SAVSBPM18 which can maintain the tetrameric state at 40°C should be stable enough for affinity purification of SBP-tagged or biotinylated proteins.

### Application of SAVSBPM18 to Purify SBP-tagged β-lactamase

**Figure 5:**
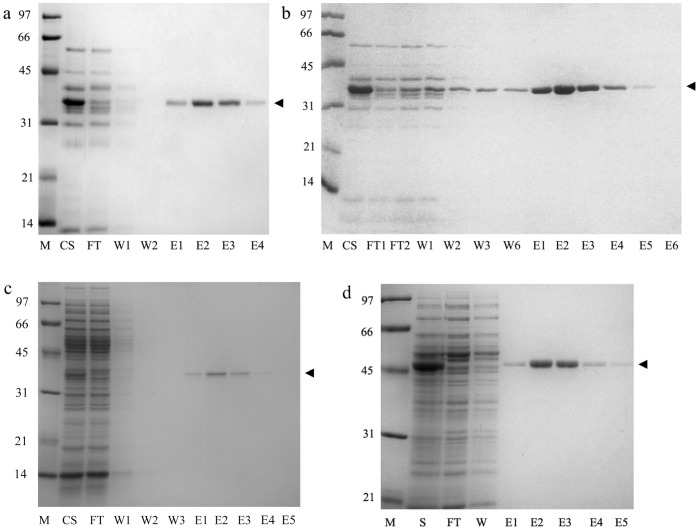
Purification of SBP tagged or biotinylated protein using SAVSBPM18-affigel. Purification of β-lactamase-SBP from the *B. subtilis* WB800[pWB980-BLA-FLSBP] culture supernatant under the column non-overloaded (panels a and c) and overloaded (panel b) conditions, respectively. The difference between samples shown in panels a and c is that sample in panel c contained externally added *E. coli* intracellular proteins to increase the background of contaminants in this sample. Purification of biotinylated maltose binding protein from a crude sample is shown in panel d. M: molecular weight markers; CS: culture supernatant containing secreted β-lactamase-SBP; S: sample of biotinylated maltose binding protein contaminated with *E. coli* intracellular proteins; FT: flow-through fraction; W: wash fraction and E: elution fraction. The positions of β-lactamase-SBP and biotinylated maltose binding protein are marked by arrowheads.

β-lactamase-SBP was used as a model protein for purification using both SAVSBPM18-affigel and wild-type streptavidin-affigel matrices. Culture supernatants containing the secreted β-lactamase-SBP were loaded to these matrices under two different conditions from below the theoretical column binding capacity to overloading the column with excess β-lactamase-SBP. The theoretical column binding capacity was expressed in terms of micrograms of β-lactamase-SBP that can bind to the matrix if all the SBP-binding sites can be fully accessible. This value was estimated based on the assumption that a streptavidin dimer (subunits A and C or subunits B and D) can bind one β-lactamase-SBP. As shown in Fig. 5a and Table 3, under the non-overloaded condition (20% of the theoretical column binding capacity), β-lactamase-SBP could be affinity purified to high purity in one step with a recovery of approximately 90% using either the wild-type streptavidin- or the SAVSBPM18-affigel matrix. In the second condition, the amounts of sample loaded exceeded the theoretical column capacity by 42–50%. Leakage of the bound β-lactamase-SBP from the SAVSBPM18-affigel column during washing was observed (Fig. 5b). However, besides the first two wash fractions which had contaminants, wash fractions 3–6 contained essentially pure β-lactamase-SBP. Therefore, fractions 3–6 were pooled together with the elution fractions to increase the recovery. Analyses of the boiled matrices which corresponded to a quarter of the total matrices in the column by SDS-PAGE and Western blot indicate that no β-lactamase-SBP could be detected (data not shown). Thus, the amounts of β-lactamase-SBP molecules retained on the column after elution are insignificant. Leakage of β-lactamase-SBP during washing was also observed with the wild-type streptavidin matrix. Under the column overloaded condition, the final recovery was around 51% for both the wild-type streptavidin and SAVSBPM18 matrices.

**Table 3:** Purification of β-lactamase-SBP using various streptavidin matrices.

Protein	Theoretical binding capacity* (µg)	Amount (µg) of β-lactamase-SBP loaded	Percent capacity (%)	Amount recovered (µg)	Percent recovery (%)
SBPM18	1102	220	20	200	91
	1102	1650	150	953**	58
wt streptavidin	1038	210	20	188	90
	1038	1475	142	753**	51

* Theoretical binding capacity is expressed in terms of the amounts (µg) of β-lactamase-SBP that can bind to the matrix. This value is estimated with the assumption that one SBP tag binds to two subunits in a tetrameric streptavidin. 1 µg of streptavidin dimer (Mr = 33,038) will bind 1.06 µg of β-lactamase-SBP (Mr = 35,030.6). New matrix was used in each binding study.

** Amount recovered is based on the total amount of pure proteins recovered in selected wash fractions and the elution fractions.

SAVSBPM18 matrix has also been successfully employed to purify β–lactamase-SBP under a low abundance condition with a high background of contaminated proteins. This challenging condition was simulated by diluting the β–lactamase-SBP containing culture supernatant with an intracellular protein extract from *E. coli*. Fig. 5c shows that highly purified β–lactamase-SBP could be separated from the large amounts of contaminants in one step on the column. To regenerate the SAVSBPM18 matrix for next cycle of purification, the column was simply washed with the wash buffer. The SAVSBPM18 matrix had been reused 8 times without any obvious loss in binding capacity.

### Application of SAVSBPM18 to Purify Biotinylated Maltose Binding Protein

SAVSBPM18-affigel could be applied to purify biotinylated maltose binding protein effectively. Over 75% of the eluted maltose binding proteins were in the second and third elution fractions (Fig. 5d). The overall recovery was approximately 90%. No biotinylated MBP could be detected from the column matrix after elution. This matrix could be easily regenerated by washing the column with the wash buffer and had been reused at least five times without any detectable loss in binding capacity. Currently, a reusable affinity matrix based on an engineered streptavidin mutein is commercially available for purification of biotinylated proteins [25]. However, this streptavidin mutein matrix cannot be applied to purify SBP tagged protein since it did not bind β-lactamase-SBP (data not shown). Consequently, SAVSBPM18 is the best choice available for the flexibility in affinity purifying biotinylated or SBP tagged proteins in high purity.

## Discussion

### Rationale Behind the Design of SAVSBPM18

Because of the high affinity and specificity of the SBP tag to streptavidin, this system has been successfully applied for one-step affinity purification of proteins [5], [7], [26], for studying protein-protein interactions [26], [27] and for developing improved tandem tags in protein studies [6], [27], [28], [29]. To make this powerful technology to be cost effective, development of an idealized streptavidin mutein, SAVSBPM18, is essential. The key to the successful development of SAVSBPM18 relies on the ability to create this desirable mutein with minimal changes in the streptavidin sequence. Structural analysis of the streptavidin-SBP complex [13] suggests that both the G48T and S27A mutations should not significantly affect SBP tag binding but should reduce the biotin binding affinity (Fig. 1). Characterization of SAVSBPM18 indeed confirms this prediction (Table 1). Another important reason to select G48T and S27A in streptavidin for mutagenesis is that these mutations are less likely to affect subunit interactions in streptavidin. These residues are not located at the subunit interface or in a loop structure (i.e. loop_7–8_) that is involved in subunit interactions [30], [31], [32]. Consequently, SAVSBPM18 should have a stable tetrameric structure in solution and this prediction was verified by the HPLC-light scattering analysis. A stable tetrameric structure is essential for tight SBP tag binding for three reasons. First, a complete binding pocket to bind either the N-terminal (GHVVEG) or C-terminal (LEHHPQG) binding segment of the SBP tag is formed by two subunits (e.g. subunits A/C or subunits B/D, Fig. 1a). Second, stable binding of a single SBP tag requires each of the N- and C-terminal binding segments of the SBP tag to bind to the corresponding binding pocket in streptavidin simultaneously to gain the avidity effect. If SAVSBPM18 dissociates into monomers, its ability to bind SBP tag will be greatly reduced. Even if SAVSBPM18 dissociates into dimers, it will still have poor ability to bind the SBP tag. This is because each subunit in these dimers does not possess a complete SBP-binding pocket. The natural tetrameric streptavidin is formed by dimerization of two dimers. Subunits A and B (or subunits C and D) have strong subunit interfacial interactions and spontaneously form the stable A/B or C/D dimers. However, formation of tetrameric streptavidin is only mediated by weaker interfacial interactions between these stable dimers. If streptavidin dissociates into dimers, these dimers are formed by subunits A/B or C/D (not the diagonally oriented subunits A/C or B/D). These dimers do not have a full binding pocket to bind SBP tag stably. Third, to generate the affinity matrix, it is vital to have only one subunit in the streptavidin tetramer to be coupled to the matrix so that all the binding sites in the tetramer can be more readily accessible. This is particularly important for SBP tag binding since one tag has to interact with two diagonally oriented subunits (A/C or B/D subunits). If the other three subunits that are not covalently coupled to the matrix have a tendency to dissociate from tetrameric streptavidin, leakage of streptavidin subunits from the matrix can possibly be observed. This will decrease the column binding capacity and the released streptavidin subunits can contaminate the purified proteins.

### Purification of SBP-tagged β-lactamase

Although the SBP tag was developed 11 years ago, no systematic study of the streptavidin column capacity in relation to the binding behaviour of the SBP-tagged proteins has been made. As illustrated in this study, overloading the column could weaken the interaction between the SBP-tagged protein and streptavidin. Leakage of the SBP-tagged protein in the wash fractions was observed. This observation, in fact, can be explained based on the binding mode of SBP to streptavidin. When the SBP-tagged proteins are in excess, only one of the two binding segments in the SBP tag is most likely to interact with a binding pocket in the tetrameric streptavidin. Since either the N- or C-terminal streptavidin binding segment alone in a SBP tag is not strong enough to allow the SBP tag to bind tightly to streptavidin [33], leakage of the bound SBP-tagged proteins can be expected. To take advantage of the full binding strength between the SBP-tagged proteins and the matrices, the amounts of SBP-tagged protein loaded should not exceed the column capacity.

### Advantage of SAVSBPM18 Over Wild-type Streptavidin in Purifying SBP-tagged and Biotinylated Proteins

Wild-type streptavidin cannot be applied to affinity purify biotinylated proteins since it binds the proteins almost irreversibly. As for SBP-tagged proteins, inability to reuse the wild-type streptavidin matrix is a major drawback. The purification cost will be very high. Furthermore, presence of biotin in the protein samples is another common complication. This often leads to lower column binding capacity and poor recovery because of the irreversible poisoning of some or all of the streptavidin molecules in the matrix if biotin in the protein samples is not removed by extensive dialysis. SBPM18 overcomes this problem and allows many rounds of reuse of the matrix.

### Advantage of the SBP Tag Over Other Tags

Many tags are available for affinity purification of proteins [34], [35]. Among these, His-tag is one of the most popular ones. Depending on the matrices used, the binding affinity of the His-tag protein in general is relatively low (K_d_ ∼ µM) [36]. Furthermore, many metal binding proteins and proteins with naturally surface exposed histidine residues can bind non-specifically to the matrix. The purity of the purified His-tag proteins subsequently is around 80% or lower. Fusion of His-tag to proteins has also been reported in some cases to interfere with protein folding [37], [38], [39], affect substrate binding [40] and cause heterogeneity of the tagged proteins [41], [42].

Besides the SBP tag, two relatively high affinity streptavidin-binding tags are available. The first one is a 15-amino-acid nano-tag (K_d_ ∼4 nM) [43]. Because of its mode of interaction with streptavidin, this tag has to be localized at the N-terminus of the recombinant protein [44]. Furthermore, a formylated methionine is required at the N-terminus for high-affinity interactions. Consequently, the recombinant proteins can only be produced from bacteria. The second tag is strep-tag III [45] which was developed on a principle similar to the SBP tag. It has two strep-tag II sequences separated by a linker and can bind to Strep-Tactin [46], an engineered streptavidin with a higher strep-tag II binding affinity (K_d_ ∼10^−6^M). This system has a drawback. Although strep-tag containing proteins can be eluted off from the matrix using desthiobiotin, the binding capacity of the column tends to decrease with use. This is because of the presence of contaminating biotin in the desthiobiotin supply. Furthermore, to achieve a better recovery of the strep-tag proteins, biotin is recommended for elution. Since Strep-Tactin binds tightly to biotin in a similar manner as wild-type streptavidin, regeneration of the column would not be feasible. In fact, the principle applied here to develop SAVSBPM18 can potentially be applied to develop improved versions of Strep-Tactin with reversible biotin binding capability.

#### Other possible applications

Development of SAVSBPM18 revolutionizes the technology in purification of SBP tagged or biotinylated proteins. Most importantly, it allows reuse of the matrix and offers the potential for the development of automated high-throughput protein purification systems. SAVSBPM18 can also be immobilized to different platforms such as protein arrays, ELISA plates, magnetic beads, biosensor chips, lab-on-a-chip micro-devices and bioreactors for different applications [47]. It is important to note that proteins (or protein libraries) tagged with other tags including His-tag [48], Halo-tag [49] and Snap-tag [50] can, in fact, be easily converted to the biotinylated format. They can then be affinity purified and immobilized using the SAVSBPM18 column and the wild-type streptavidin based protein array, respectively. Furthermore, horseradish peroxidase or fluorescent dye conjugated SAVSBPM18 can potentially be applied for probing SBP-tagged or biotinylated proteins in Western blots and biotinylated DNA/RNA in Southern/Northern blots. Reprobing of these blots can be done easily since SAVSBPM18 can be stripped off gently. In certain studies, biotin-free culture media are needed to examine the biotin effects on transport, cell growth and protein production. Traditionally, these media have to pass through avidin or streptavidin matrix to remove biotin [51]. With the development of SAVSBPM18, these media can be prepared in a much more cost-effective manner. The SAVSBPM18 matrix can also be applied to prepare biotin-free desthiobiotin for the use in the Strep-Tacin system.

## References

[1] Pouny Y, Weitzman C, Kaback HR (1998) *In vitro* biotinylation provides quantitative recovery of highly purified active lactose permease in a single step. Biochemistry 37: 15713–15719. 9843376 10.1021/bi981519z

[2] Julien M, Kajiji S, Kaback RH, Gros P (2000) Simple purification of highly active biotinylated P-glycoprotein: enantiomer-specific modulation of drug-stimulated ATPase activity. Biochemistry 39: 75–85. 10625481 10.1021/bi991726e

[3] Elia G (2010) Protein Biotinylation. Current Protocols in Protein Science: John Wiley & Sons, Inc. 3.6.1–3.6.21. 10.1002/0471140864.ps0306s60 20393976

[4] Cull MG, Schatz PJ (2000) Biotinylation of proteins *in vivo* and *in vitro* using small peptide tags. Methods in enzymology 326: 430–440. 11036656 10.1016/s0076-6879(00)26068-0

[5] Keefe AD, Wilson DS, Seelig B, Szostak JW (2001) One-step purification of recombinant proteins using a nanomolar-affinity streptavidin-binding peptide, the SBP-Tag. Protein Expr Purif 23: 440–446. 11722181 10.1006/prep.2001.1515

[6] Van Leene J, Witters E, Inze D, De Jaeger G (2008) Boosting tandem affinity purification of plant protein complexes. Trends Plant Sci 13: 517–520. 18771946 10.1016/j.tplants.2008.08.002

[7] Kobayashi T, Morone N, Kashiyama T, Oyamada H, Kurebayashi N, et al (2008) Engineering a novel multifunctional green fluorescent protein tag for a wide variety of protein research. PLoS ONE 3: e3822. 19048102 10.1371/journal.pone.0003822 PMC2585475

[8] Huang X, Zhang XE, Zhou YF, Zhang ZP, Cass AE (2007) Construction of a high sensitive *Escherichia coli* alkaline phosphatase reporter system for screening affinity peptides. Journal of biochemical and biophysical methods 70: 435–439. 17156847 10.1016/j.jbbm.2006.10.006

[9] Green NM (1990) Avidin and streptavidin. Methods Enzymol 184: 51–67. 2388586 10.1016/0076-6879(90)84259-j

[10] Wu SC, Qureshi MH, Wong SL (2002) Secretory production and purification of functional full-length streptavidin from *Bacillus subtilis* Protein Expr Purif. 24: 348–356. 10.1006/prep.2001.1582 11922750

[11] Wu SC, Yeung JC, Duan Y, Ye R, Szarka SJ, et al (2002) Functional production and characterization of a fibrin-specific single-chain antibody fragment from *Bacillus subtilis* : Effects of molecular chaperones and a wall-bound protease on antibody fragment production. Applied and Environmental Microbiology 68: 3261–3269. 12089002 10.1128/AEM.68.7.3261-3269.2002 PMC126797

[12] Wu SC, Wong SL (2002) Engineering of a *Bacillus subtilis* strain with adjustable levels of intracellular biotin for secretory production of functional streptavidin. Applied and Environmental Microbiology 68: 1102–1108. 11872456 10.1128/AEM.68.3.1102-1108.2002 PMC123784

[13] Barrette-Ng IH, Wu SC, Tjia WM, Wong SL, Ng KK (2013) The structure of the SBP-Tag-streptavidin complex reveals a novel helical scaffold bridging binding pockets on separate subunits. Acta crystallographica Section D, Biological crystallography 69: 879–887. 23633599 10.1107/S0907444913002576 PMC3640474

[14] Wu SC, Wong SL (2005) Engineering soluble monomeric streptavidin with reversible biotin binding capability. J Biol Chem 280: 23225–23231. 15840576 10.1074/jbc.M501733200

[15] Qureshi MH, Yeung JC, Wu SC, Wong SL (2001) Development and characterization of a series of soluble tetrameric and monomeric streptavidin muteins with differential biotin binding affinities. J Biol Chem 276: 46422–46428. 11584006 10.1074/jbc.M107398200

[16] Klumb LA, Chu V, Stayton PS (1998) Energetic Roles of Hydrogen Bonds at the Ureido Oxygen Binding Pocket in the Streptavidin-Biotin Complex. Biochemistry 37: 7657–7663. 9601024 10.1021/bi9803123

[17] Hofmann K, Wood SW, Brinton CC, Montibeller JA, Finn FM (1980) Iminobiotin affinity columns and their application to retrieval of streptavidin. Proc Natl Acad Sci USA 77: 4666–4668. 6933515 10.1073/pnas.77.8.4666 PMC349906

[18] Harding SE, Jumel K (2001) Light scattering. Curr Protoc Protein Sci Chapter 7: Unit 7.8. 10.1002/0471140864.ps0708s11 18429201

[19] Thomsen R, Christensen MH (2006) MolDock: a new technique for high-accuracy molecular docking. J Med Chem 49: 3315–3321. 16722650 10.1021/jm051197e

[20] Krieger E, Koraimann G, Vriend G (2002) Increasing the precision of comparative models with YASARA NOVA–a self-parameterizing force field. Proteins 47: 393–402. 11948792 10.1002/prot.10104

[21] Stayton PS, Freitag S, Klumb LA, Chilkoti A, Chu V, et al (1999) Streptavidin-biotin binding energetics. Biomol Eng 16: 39–44. 10796983 10.1016/s1050-3862(99)00042-x

[22] Freitag S, Le Trong I, Klumb L, Stayton PS, Stenkamp RE (1997) Structural studies of the streptavidin binding loop. Protein Sci 6: 1157–1166. 9194176 10.1002/pro.5560060604 PMC2143724

[23] Wilmot CM, Thornton JM (1988) Analysis and prediction of the different types of beta-turn in proteins. J Mol Biol 203: 221–232. 3184187 10.1016/0022-2836(88)90103-9

[24] Williams RW, Chang A, Juretic D, Loughran S (1987) Secondary structure predictions and medium range interactions. Biochimica et biophysica acta 916: 200–204. 3676331 10.1016/0167-4838(87)90109-9

[25] Kopetzki E, Muller R, Engh R, Schmitt U, Deger A, et al. (2001) Recombinant inactive core streptavidin mutants. US 6312916 B1.

[26] Kim JH, Chang TM, Graham AN, Choo KH, Kalitsis P, et al (2010) Streptavidin-Binding Peptide (SBP)-tagged SMC2 allows single-step affinity fluorescence, blotting or purification of the condensin complex. BMC Biochem 11: 50. 21194474 10.1186/1471-2091-11-50 PMC3022668

[27] Li Y, Franklin S, Zhang MJ, Vondriska TM (2011) Highly efficient purification of protein complexes from mammalian cells using a novel streptavidin-binding peptide and hexahistidine tandem tag system: application to Bruton's tyrosine kinase. Protein Sci 20: 140–149. 21080425 10.1002/pro.546 PMC3047070

[28] Burckstummer T, Bennett KL, Preradovic A, Schutze G, Hantschel O, et al (2006) An efficient tandem affinity purification procedure for interaction proteomics in mammalian cells. Nat Methods 3: 1013–1019. 17060908 10.1038/nmeth968

[29] Li Y (2011) The tandem affinity purification technology: an overview. Biotechnol Lett 33: 1487–1499. 21424840 10.1007/s10529-011-0592-x

[30] Hendrickson WA, Pahler A, Smith JL, Satow Y, Merritt EA, et al (1989) Crystal structure of core streptavidin determined from multiwavelength anomalous diffraction of synchrotron radiation. Proc Natl Acad Sci USA 86: 2190–2194. 2928324 10.1073/pnas.86.7.2190 PMC286877

[31] Weber PC, Ohlendorf DH, Wendoloski JJ, Salemme FR (1989) Structural origins of high-affinity biotin binding to streptavidin. Science 243: 85–88. 2911722 10.1126/science.2911722

[32] Sano T, Cantor CR (1995) Intersubunit contacts made by tryptophan 120 with biotin are essential for both strong biotin binding and biotin-induced tighter subunit association of streptavidin. Proc Natl Acad Sci USA 92: 3180–3184. 7724536 10.1073/pnas.92.8.3180 PMC42129

[33] Wilson DS, Keefe AD, Szostak JW (2001) The use of mRNA display to select high-affinity protein-binding peptides. Proc Natl Acad Sci USA 98: 3750–3755. 11274392 10.1073/pnas.061028198 PMC31124

[34] Arnau J, Lauritzen C, Petersen GE, Pedersen J (2006) Current strategies for the use of affinity tags and tag removal for the purification of recombinant proteins. Protein Expr Purif 48: 1–13. 16427311 10.1016/j.pep.2005.12.002

[35] Lichty JJ, Malecki JL, Agnew HD, Michelson-Horowitz DJ, Tan S (2005) Comparison of affinity tags for protein purification. Protein Expr Purif 41: 98–105. 15802226 10.1016/j.pep.2005.01.019

[36] Nieba L, Nieba-Axmann SE, Persson A, Hamalainen M, Edebratt F, et al (1997) BIACORE analysis of histidine-tagged proteins using a chelating NTA sensor chip. Anal Biochem 252: 217–228. 9344407 10.1006/abio.1997.2326

[37] Pekrun K, Petry H, Jentsch KD, Moosmayer D, Hunsmann G, et al (1995) Expression and characterization of the reverse transcriptase enzyme from type 1 human immunodeficiency virus using different baculoviral vector systems. Eur J Biochem 234: 811–818. 8575439 10.1111/j.1432-1033.1995.811_a.x

[38] Ledent P, Duez C, Vanhove M, Lejeune A, Fonze E, et al (1997) Unexpected influence of a C-terminal-fused His-tag on the processing of an enzyme and on the kinetic and folding parameters. FEBS Lett 413: 194–196. 9280280 10.1016/s0014-5793(97)00908-3

[39] Halliwell CM, Morgan G, Ou CP, Cass AE (2001) Introduction of a (poly)histidine tag in L-lactate dehydrogenase produces a mixture of active and inactive molecules. Anal Biochem 295: 257–261. 11488630 10.1006/abio.2001.5182

[40] Slessor KE, Stok JE, Cavaignac SM, Hawkes DB, Ghasemi Y, et al (2010) Cineole biodegradation: molecular cloning, expression and characterisation of (1R)-6beta-hydroxycineole dehydrogenase from *Citrobacter braakii* . Bioorg Chem 38: 81–86. 20089292 10.1016/j.bioorg.2009.12.003

[41] Narmandakh A, Bearne SL (2010) Purification of recombinant mandelate racemase: improved catalytic activity. Protein Expr Purif 69: 39–46. 19589387 10.1016/j.pep.2009.06.022

[42] Khan F, He M, Taussig MJ (2006) Double-hexahistidine tag with high-affinity binding for protein immobilization, purification, and detection on ni-nitrilotriacetic acid surfaces. Anal Chem 78: 3072–3079. 16642995 10.1021/ac060184l

[43] Lamla T, Erdmann VA (2004) The Nano-tag, a streptavidin-binding peptide for the purification and detection of recombinant proteins. Protein Expr Purif 33: 39–47. 14680960 10.1016/j.pep.2003.08.014

[44] Perbandt M, Bruns O, Vallazza M, Lamla T, Betzel C, et al (2007) High resolution structure of streptavidin in complex with a novel high affinity peptide tag mimicking the biotin binding motif. Proteins 67: 1147–1153. 17377987 10.1002/prot.21236

[45] Junttila MR, Saarinen S, Schmidt T, Kast J, Westermarck J (2005) Single-step Strep-tag purification for the isolation and identification of protein complexes from mammalian cells. Proteomics 5: 1199–1203. 15761952 10.1002/pmic.200400991

[46] Voss S, Skerra A (1997) Mutagenesis of a flexible loop in streptavidin leads to higher affinity for the Strep-tag II peptide and improved performance in recombinant protein purification. Protein Engineering 10: 975–982. 9415448 10.1093/protein/10.8.975

[47] Xia H, Murray K, Soper S, Feng J (2012) Ultra sensitive affinity chromatography on avidin-functionalized PMMA microchip for low abundant post-translational modified protein enrichment. Biomed Microdevices 14: 67–81. 21915645 10.1007/s10544-011-9586-7

[48] Reichel A, Schaible D, Al Furoukh N, Cohen M, Schreiber G, et al (2007) Noncovalent, site-specific biotinylation of histidine-tagged proteins. Analytical chemistry 79: 8590–8600. 17953454 10.1021/ac0714922

[49] Heal WP, Wright MH, Thinon E, Tate EW (2012) Multifunctional protein labeling via enzymatic N-terminal tagging and elaboration by click chemistry. Nat Protoc 7: 105–117. 10.1038/nprot.2011.425 22193303

[50] Iversen L, Cherouati N, Berthing T, Stamou D, Martinez KL (2008) Templated protein assembly on micro-contact-printed surface patterns. Use of the SNAP-tag protein functionality. Langmuir 24: 6375–6381. 18484753 10.1021/la7037075

[51] Waller JR, Anderson JK, Ulmer DC (1984) Use of avidin to prepare biotin-free culture media. Analytical biochemistry 141: 189–193. 6496927 10.1016/0003-2697(84)90444-5

[52] Qureshi MH, Wong SL (2002) Design, production, and characterization of a monomeric streptavidin and its application for affinity purification of biotinylated proteins. Protein Expr Purif 25: 409–415. 12182820 10.1016/s1046-5928(02)00021-9

